# HMGN1 loss sensitizes lung cancer cells to chemotherapy

**DOI:** 10.1038/s41598-024-60352-8

**Published:** 2024-05-06

**Authors:** Xianli Wu, Geqi Cai, Jing Feng, Wenchu Lin

**Affiliations:** 1 Department of Pathology and Pathophysiology, School of Basic Medicine, Anhui Medical University, Hefei, 230032 Anhui China; 2 School of Laboratory Medicine and Biotechnology, Southern Medical University, Guangzhou, Guangdong China; 3 The Second Affiliated Hospital, School of Medicine, The Chinese University of Hong Kong, Shenzhen & Longgang District People’s Hospital of Shenzhen, Shenzhen, 518172 China

**Keywords:** Lung cancer, Tumour biomarkers, Cancer, Computational biology and bioinformatics, Molecular biology, Biomarkers, Molecular medicine, Oncology

## Abstract

The high mobility group nucleosome binding (HMGN) family, constitutes a large family of non-histone protein family known to bind the acidic patch of the nucleosomes with various key cellular functions. Several studies have highlighted the pivotal roles of HMGNs in the pathogenic process of various cancer types. However, the roles of HMGN family in lung adenocarcinoma (LUAD) have not been fully elucidated. Herein, integrative analyses of multiple-omics data revealed that HMGNs frequently exhibit dysregulation in LUAD. Subsequent analysis of the clinical relevance of HMGN1 demonstrated its association with poor prognosis in LUAD and its potential as a diagnostic marker to differentiate LUAD from healthy controls. Additionally, functional enrichment analysis suggested that HMGN1 was mainly involved in DNA repair. To corroborate these findings, cellular experiments were conducted, confirming HMGN1’s crucial involvement in homologous recombination repair and its potential to enhance the sensitivity of LUAD cells to standard chemotherapeutic drugs. This study proposes HMGN1 as a novel prognostic biomarker and a promising target for chemotherapy in lung adenocarcinoma.

## Introduction

Lung cancer, recognized as the leading cause of cancer-related deaths globally^1^, is primarily categorized into non-small-cell lung carcinoma (NSCLC) and small-cell lung carcinoma (SCLC), with NSCLC further subdivided into lung adenocarcinoma (LUAD), squamous cell carcinoma, and large cell carcinoma based on pathological classification^2^. Notably, LUAD is the most prevalent subtype, accounting for 40% of all new lung cancer cases diagnosed annually worldwide. Despite considerable advancements in therapeutic strategies for LUAD in recent decades, the long‐term prognosis for patients remains a significant clinical challenge^3^. Consequently, the development of early diagnostic methods and the implementation of effective targeted therapies are imperative for this devastating disease.

DNA, the carrier of storing and transmitting genetic information, is organized into chromatin in eukaryotic cells through stably interactions with histone and non-histone proteins. The chromatin structure is a dynamic entity, continuously modified by nuclear factors, which compact the genome for efficient accommodation within the nucleus^4^. Critical DNA-dependent events, including transcription, replication, and DNA repair, are intricately linked to the chromatin, necessitating precise spatial and temporal regulation of its structure^5^. High mobility group N (HMGN), a group of chromatin-binding architectural proteins, binds specifically to nucleosomes without preference for any DNA sequence^6^. The association of HMGN with nucleosomes impedes histone H1 binding, thereby influencing chromatin accessibility and remodeling^7^. To date, five HMGN family members have been discovered in human genome. All of them possess a positively charged nucleosome binding domain, a bipartite nuclear localization signal (NLS), and an acidic C-terminal chromatin regulatory domain^8^. HMGN1 and HMGN2 are ubiquitously expressed in mammalian cells, while HMGN3, 4, and 5 have more restricted expression patterns^7^. The interaction of HMGN proteins with nucleosomes destabilizes the high-order chromatin structure, modulates the pattern of histone post-translational modification, and enhances the accessibility of non-histone proteins such as transcription factors, thereby significantly influencing cellular phenotypes^9^.

Increasing evidence has demonstrated that HMGN genes are implicated in development, immunological processes, and the etiology of disease, including cancer^10^. For example, appropriate expression levels of HMGN family proteins are crucial in both maintenance of the pluripotent identity of stem cells and facilitating cellular differentiation during embryogenesis^11,12^. HMGN1, acting as an alarmin, fosters antitumor immunity by inducing maturation in human dendritic cells via Toll-like receptor 4 (TLR-4)^13^. Additionally, high HMGN1 levels correlate with increased peri-tumor infiltration of lymphocytes in Her2-positive breast cancer^14^. Moreover, recent studies in head and neck carcinoma have linked elevated cytoplasmic HMGN1 levels with increasing tumor-infiltrating lymphocytes^15^, indicating potential immunotherapeutic applications. HMGN2 promotes breast cancer progression by facilitating STAT5 access to the promoter region of its targets^16^. Elevated HMGN4 expression is associated with high grade tumors and poor outcomes in hepatocellular carcinoma^17^. Despite growing evidence of aberrant HMGN family gene expression across multiple tumor lineages, their specific roles in lung adenocarcinoma, the predominant type of lung cancer, remain largely unexplored.

Given the crucial role of HMGN proteins in chromatin de-condensation and posttranslational modifications in histone tails, which are integral to DNA damage repair, it is plausible that HMGN family protein may be involved in DNA damage response. Indeed, studies show that HMGN1^-/-^ mice and cells are hypersensitive to UV radiation due to impaired accessibility to UV-damaged sites in chromatin^18^. Loss of HMGN1 also impairs ionizing radiation (IR)-induced ATM auto-phosphorylation and the activation of several ATM targets in mouse embryonic fibroblasts^19^. However, the mechanisms by which HMGN proteins modulate DNA repair processes are yet to be fully understood, warranting systematic analysis and exploration.

Prior research has indicated aberrant expressions of HMGNs and their prognostic value in some members of HMGN family^20^. Nevertheless, the role of distinct HMGN family members in the development and progression of lung adenocarcinoma have not been clearly defined. Through comprehensive analyses of multi-level omics data, this study investigated the expression and prognostic significance of HMGNs in lung adenocarcinoma. We discovered that HMGN1 was up-regulated in LUAD and its expression was associated with clinical outcomes. Function enrichment analysis further revealed a strong association between HMGN1 expression six DNA damage repair pathways. Finally, the roles of HMGN1 in the ATR-ChK1 and DNA double-strand break (DSB) repair pathways was manifested in vitro in lung adenocarcinoma cells.

## Materials and methods

### Data collection

#### LUAD cohort

Clinical information and RNA-seq data of LUAD patients were sourced from the TCGA database^21^ (https://portal.gdc.cancer.gov). Additionally, gene chips numbered GSE11969, GSE18842, GSE10072, GSE13213, GSE19804, and GSE116959 were selected from the GEO database (http://www.ncbi.nlm.nih.gov/geo/)^22^.

#### Human protein atlas

Human Protein Atlas database (http://www.proteinatlas.org/) were utilized to analyze HMGNs expression in LUAD tissues compared to adjacent normal tissues at the protein level^23^.

#### Ualcan dataset

Ualcan dataset (http://ualcan.path.uab.edu) was employed to examine the protein expression of HMGNs in LUAD tissues versus normal tissues from Clinical Proteomic Tumor Analysis Consortium (CPTAC). Additionally, this dataset provided DNA promoter methylation profiles of HMGN family in LUAD from TCGA^24^.

#### cBioportal

Mutation profiles of HMGN family genes were analyzed by a standard processing pipeline in the cBioPortal (https://www.cbioportal.org)^25^.

### Weighted gene co-expression network analysis (WGCNA)

WGCNA was used to analysis gene association patterns in LUAD patients from TCGA. Initially, a correlation coefficient was calculated between two genes based on their expression patterns, followed by the construction of a gene network based on these coefficients. Optimal soft thresholding was determined using the 'pick soft threshold' function to categorize genes with high correlation into modules. The module containing HMGN1 was identified as 'brown', and genes in this module were subjected to GO analysis using the DAVID dataset (https://david.ncifcrf.gov/summary.jsp). The top 8 channels with P value less than 0.05 were selected^26^.

### Gene set enrichment analysis (GSEA)

GSEA was conducted using the clusterProfiler (version 4.4.4) and enrichplot (version 1.16.1) R packages. RNA-Seq data from 516 lung adenocarcinoma specimens and pre-defined gene sets based on the Human MSigDB v2022.1 were used to identify signatures associated with HMGN1 expression in LUAD^27^.

### Cell culture

Human lung adenocarcinoma cell lines A549 and PC9 were kindly provided by Dr. Matthew Meyerson at Dana-Farber Cancer Institute, USA. LUAD cell lines A549 and PC9 were maintained in RPMI-1640 containing 10% fetal bovine serum and 1% penicillin/streptomycin in a humidified incubator at 37 °C with 5% CO_2_. Regular testing for bacterial and mycoplasma contamination was conducted.

### RNA interference and stable cell line construction

HMGN1 siRNAs sequences, referenced from relevant literature^28,29^ and synthesized by General Biosystems (Hefei, China). Cells at 50% density confluency in 6-well plates were transfected with HMGN1 siRNAs or NC-siRNA using the Effectene transfection agent, following the manufacturer’s instruction. After 48 h of culture, cells were harvested for further analysis. For stable cell line construction, the short hairpin RNA (shRNA) oligonucleotides of HMGN1 were synthesized based on siRNA sequence and cloned into the pLKO.1 vector (Sigma). Resulting constructs were packaged into lentivirus for transduction into LUAD cancer cells. Stable cell lines were established by puromycin selection over one week.

### RNA preparation and quantitative real-time PCR

Total RNA was extracted from cultured cells using the Trizol-up plus RNA kit (Thermo Scientific), following the manufacturer’s protocol. cDNA synthesis was performed using the Transcriptor First Strand cDNA Synthesis Kit (Roche). Real-time quantitative PCR (RT-qPCR) was conducted using ChamQ SYBR qPCR Master Mix (Vazyme) in a Roche LC96 Real-Time PCR System. mRNA expression levels were calculated using the 2^−ΔΔCt^ method. The primers used were as follows^30–32^:

HMGN1-F: 5′-TGCAAACAAAAGGGAAAAGG-3′

HMGN1-R: 5′ CATCAGAGGCTGGACTCTCC -3′

β-actin-F: 5′-CATGTACGTTGCTATCCAGGC-3′.

β-actin-R: 5′-CTCCTTAATGTCACGCACGA-3′.

RAD51-F: 5′-CAACCCATTTCACGGTTAGAGC-3′

RAD51-R: 5′-TTCTTTGGCGCATAGGCAACA-3′

### Western blot

Standard Western blotting protocols were followed as previously described^32^. Primary antibodies used included RAD51 (1:1000, Abcam ab133534), γH2AX (1:1000, CST 2577), p-CHK1 (1:1000, Ser317, CST 12302), CHK1 (1:1000, CST 2G1D5), β-actin (1:1000, TransGen HC201-02), p-RPA2 (1:1000,NOVUS,Ser4, Ser8, NBP1-23017), RPA2 (1:1000, Abcam ab2175), HMGN1 (1:1000, proteintech 11695-1-AP). Secondary antibodies were Rabbit IgG (1:3000, CST 7074) and mouse IgG (1:3000, CST 7076).

### Cell viability and clonogenic assay

Standard cell viability and clonogenicassay protocols were followed as previously described^31^. Cells were exposed to PBS or 2 mM HU for 2 h or 4 mM HU for 4 h. After the cells was replaced with fresh media and continued to culture for 48 h for cell viability assay using the CellTiter-Glo luminescent kit according to the manufacturer’s instructions or in six-well plates until colonies reached appropriate size (approximate 7–10 days). Cells were fixed with methanol for 5–10 min, then stained with 0.1% crystal violet solution for 15 min. After rinsing and drying, colonies were photographed. Cisplatin was administered as indicated concentrations for 48 h and the cell viability was determined using the CellTiter-Glo assay.

### Apoptotic assay

Standard apoptotic assay protocols were followed as previously described^33^. Apoptosis in cultured cells treated with PBS or indicated drugs was assessed by a FACS Calibur (Sony Biotechnology, San Jose, CA, USA) using a PI/Annexin V-FITC kit (BD Pharmingen, San Diago, CA, USA).

### Gamma-H2AX immunostaining assay

Following drug administration, cells were prepared as previously described^34^). After blocking, cells were incubated with Gamma-H2AX antibody followed by Alexa Fluor 488 conjugated secondary antibodies. Images were acquired using an upright fluorescent microscope. Cells exhibiting more than 5 foci per cell were classified as positive.

### Statistical analysis

All in vitro analyses were replicated at least three times. *P*-value < 0.05 was considered statistically significant. Data were analyzed using two-tailed unpaired Student’s t tests with GraphPad Prism software.

## Results

### Expression levels of HMGNs in LUAD

**Figure 1:**
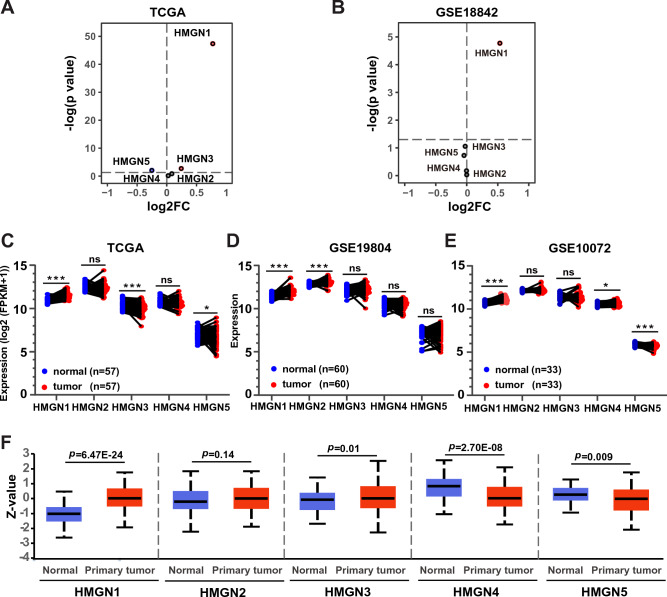
Aberrant expression of HMGN family in lung adenocarcinoma. (**A**) Volcano plots illustrating differential HMGN expression in lung adenocarcinoma versus normal tissues from the TCGA database (**A**) and GSE18842 (**B**). Expression values were presented as log_2_ (FPKM + 1). Fold changes were presented as log2 (fold change). (**C**–**E**) Transcriptional expression of HMGNs in 57 paired lung adenocarcinoma and adjacent normal tissues from TCGA (**C**); 60 pairs from GSE19804 (**D**) and 33 pairs from GSE10072 (**E**). *p < 0.05, **p < 0.01, ***p < 0.001. ns: not statistically significant. (**F**) Protein expression levels of HMGNs in lung cancer versus normal lung tissues from the CPTAC dataset.

To investigate the deregulation of HMGNs in lung adenocarcinoma (LUAD), the RNA-sequencing data of five HMGN members in LUAD and normal lung tissues were extracted from the TCGA database and two GSE data sets and analyzed. Volcano plots analysis revealed an upregulation of HMGN1 (Fig. 1A). The up-regulation of HMGN1 was confirmed in the GSE18842 and other data set (Fig. 1B, Supplementary Fig. 1A and B), while HMGN3 and HMGN5 did not exhibit a similar trend. Analysis of gene expression of LUAD and matched adjacent non-tumor tissues from three cohorts showed consistent up-regulation of HMGN1 in LUAD across all cohorts, with HMGN5 down-regulated in two of them (Fig. 1C–E). Finally, pan-cancer analysis across various cancers, including breast, colon, liver, and stomach cancers, also indicated high expression of HMGN1 (Supplementary Fig. 1C).

Protein expression of HMGNs in LUAD was then assessed using the Clinical Proteomic Tumor Analysis Consortium (CPTAC) mass-spectrometry-based proteomics dataset. The results were in line with mRNA expression data, showing increased protein levels of HMGN1 and decreased levels of HMGN4/5 in LUAD (Fig. 1F). Additionally, the Human Protein Atlas was used to corroborate these findings at the protein level (Supplementary Fig. 1B). In short, these findings indicate that abnormal expression of HMGN family members is associated with patients with LUAD.

### Genetic and epigenetic alterations of HMGNs in LUAD

**Figure 2:**
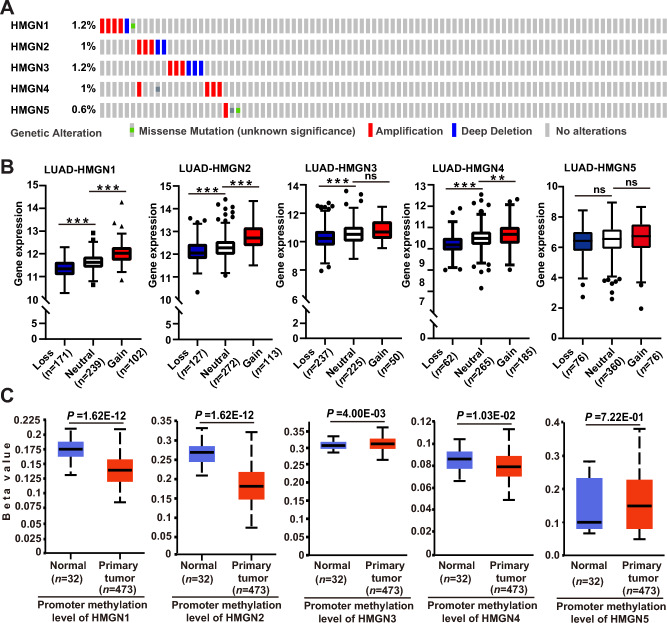
Genetic and epigenetic alterations of HMGN family in lung adenocarcinoma. (**A**) OncoPrint from the cBioPortal database showing the distribution and proportion of samples with genetic alterations in HMGN genes in LUAD. (**B**) Correlation between copy number variation and gene expression variation for HMGNs in LUAD. (**C**) The contribution of promoter methylation level of HMGNs to the expression of corresponding genes in LUAD. *p < 0.05, **p < 0.01,***p < 0.001, ns: not statistically significant.

The aberrant expression of HMGNs might be due to genetic alterations that occur in coding regions and dysregulation of epigenetics at the promoter region. Therefore, we characterized the mutation patterns of HMGNs in LUAD using the cBioPortal online tool. In general, the mutation rates of HMGNs are relatively low. HMGN genes were altered in 25 of 507 specimens (5%). Two alterations were simultaneously detected in almost 1/20 of the samples (Fig. 2A). Since genomic structural variations are the most genetic alterations in LUAD, the MEXPRESS online tools were applied to assess the transcriptional consequences of HMGN copy number variation (CNV). As shown in Fig. 2B, a significantly greater percentage of copy number gain and losses of HMGN1/2/3/4/5 in LUAD was observed. Increasing evidence has shown that DNA methylation level at promoter region is a critical factor determining transcription. Thereby the promoter methylation of HMGNs in LUAD was analyzed using the UALCAN portal. As shown in Fig. 2C, reduced methylation levels of HMGN1/2/4 were found in primary lung tumors. And promoter methylation of HMGN3/5 were up-regulated in tumor. These results implied that HMGNs gene were mutated infrequently, and the deregulation of HMGNs might be at least partly due to copy number variation and abnormal promoter methylation.

### Potential of HMGN1 as a biomarker and prognosis in LUAD

**Figure 3:**
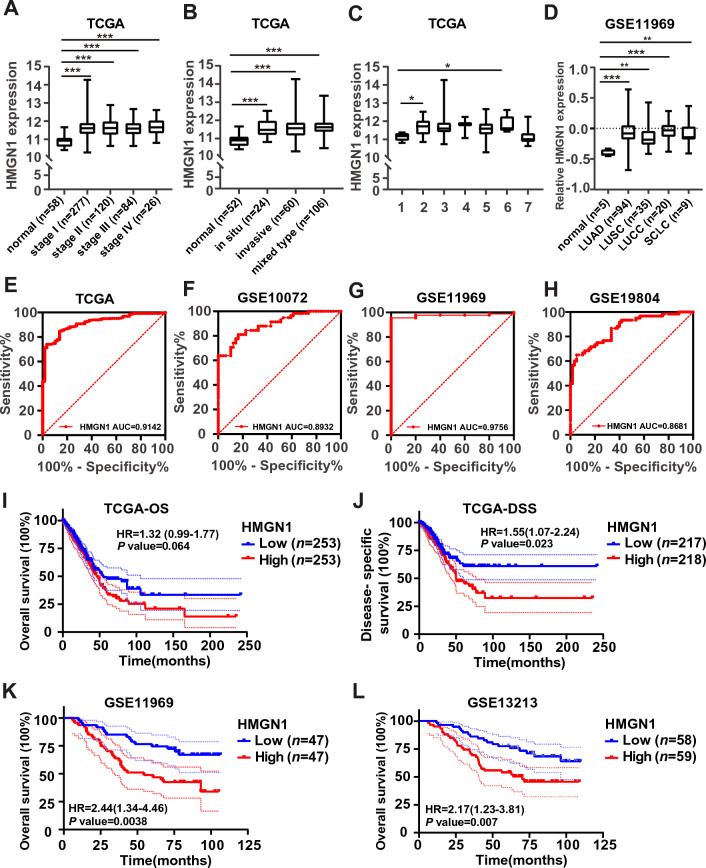
Clinical significance of HMGN1 expression in lung adenocarcinoma. (**A**) Boxplot depicting HMGN1 expression association with tumor stages in the TCGA dataset. (**B**) Boxplot illustrating the association between HMGN1 expression and tumor clinicopathological classification in the TCGA dataset. (**C**) Boxplot showing HMGN1 expression in different invasive tumor subtypes from the TCGA database, with types 1–7 representing various subtypes: 1, lung bronchioloalveolar carcinoma mucinous; 2, lung bronchioloalveolar carcinoma nonmucinous; 3, lung acinar adenocarcinoma; 4, lung micropapillary adenocarcinoma; 5, lung papillary adenocarcinoma; 6, lung solid pattern predominant adenocarcinoma; 7, mucinous (colloid) carcinoma. (**D**) Boxplot demonstrating HMGN1 expression in different lung cancer subtypes from the GSE11969 dataset. *p < 0.05, **p < 0.01, ***p < 0.001, ns: not statistically significant. (**E**–**H**) Receiver operating characteristic curve (ROC) analysis of HMGN1 expression in LUAD from TCGA (**E**) and three GEO datasets (**F**–**H**). (**I**–**J**) Kaplan–Meier curves for overall survival and disease-specific survival in LUAD patients, associated with HMGN1 expression. (**K**–**L**) Overall survival analysis of HMGN1 in LUAD from two GEO datasets.

We selected HMGN1 for subsequent assessment of prognostic values and function enrichment analysis in LUAD since HMGN1 displays most prominent changes in expression, copy number variation promoter methylation. First, the relationship between HMGN1 mRNA levels and clinicopathological subgroups of LUAD patients was evaluated in the TCGA lung cancer and GSE11969 cohorts. As shown in Fig. 3A, the expression of HMGN1 in all tumor stages (I–IV) was significantly higher than that in normal group. In addition, the expression of HMGN1 in different subtypes of LUAD was significantly higher than that in normal group (Fig. 3B and C). Furthermore, HMGN1 expression in four major subtypes of lung cancer was significantly higher than normal control (Fig. 3D). However, no difference was seen between different tumor stages/subtypes (Fig. 3B–D).

To further seek the possibility of HMGN1 as a biomarker, we analyzed the diagnostic efficiency of HMGN1 expression in discriminating LUAD patients from healthy individuals using receiver operating characteristic (ROC) curves from TCGA and GEO datasets. The data showed that HMGN1 had a high area under the curve (AUC = 0.91) value (Fig. 3E). Similar high AUC values were observed in other three lung cancer cohorts (AUC = 0.89 in GSE10072, AUC = 0.98 in GSE11969, and AUC = 0.87 in GSE19804) (Fig. 3F–H). And we analyzed the diagnostic efficiency of HMGN1 expression in discriminating LUAD patients from other lung cancer using ROC curves from GEO dataset. The data showed that HMGN1 had no high area under the curve value (Supplementary Fig. 2A). To date, the potential prognostic value of HMGN family members remains unclear. Therefore, the prognostic significance of HMGN1 mRNA expression were evaluated by using three public available datasets. Kaplan–Meier (KM) curve analysis showed that HMGN1 expression was associated with overall survival (OS, Fig. 3I and Supplementary Fig. 2C) and disease-specific survival (DSS, Fig. 3J) in LUAD patients in the TCGA cohort. However, the association of HMGN1 mRNA expression with progression free survival and disease free survival in LUAD was also seen not significantly in TCGA cohort (Supplementary Fig. 2B).Moreover the association of high HMGN1 mRNA expression with worse survival in LUAD was also seen in two other cohorts (Fig. 3K and L). Furthermore, the univariate and multivariate Cox analyses were performed to investigate the relationship between HMGN1 expression and OS or DSS. As indicated in Tables 1 and 2, the analysis shown that T stage, N stage, HMGN1 expression were associated with OS and DSS in LUAD patients. These results indicated that the expression of HMGN1 might be helpful in the prognosis of LUAD patients.

**Table 1:** Univariate and multivariate analysis of risk score and patient overall survival.

Variables	Univariate analysis			Multivariate analysis		
	Hazard Ratio	95% CI	*P* value	Hazard Ratio	95% CI	*P* value
HMGN1 High vs Low	1.32	0.98–1.77	0.064	1.39	1.03–1.87	0.029
Gender Female vs Male	0.96	0.72–1.28	0.772	0.95	0.71–1.27	0.73
M stage M0 or MX vs M1	0.99	0.72–1.36	0.928	0.95	0.69–1.31	0.766
N stage N0 or N1 or NX vs N2 or N3	0.46	0.32–0.65	0	0.52	0.36–0.75	0
T stage T1 or T2 or TX vs T3 or T4	0.46	0.32–0.67	0	0.51	0.35–0.76	0.001

**Table 2:** Univariate and multivariate analysis of risk score and patient disease special survival.

Variables	Univariate analysis			Multivariate analysis		
	Hazard Ratio	95%CI	*P* value	Hazard Ratio	95%CI	*P* value
HMGN1 High vs Low	1.58	1.08–2.31	0.02	1.57	1.07–2.3	0.021
Gender Female vs Male	1.06	0.73–1.54	0.763	1.02	0.7–1.48	0.921
M stage M0 or MX vs M1	0.85	0.57–1.26	0.41	0.83	0.56–1.24	0.364
N stage N0 or N1 or NX vs N2 or N3	0.44	0.28–0.7	0.001	0.49	0.31–0.79	0.003
T stage T1 or T2 or TX vs T3 or T4	0.46	0.28–0.76	0.003	0.49	0.29–0.81	0.006

### Functional enrichment analysis of the HMGN1 in LUAD

**Figure 4:**
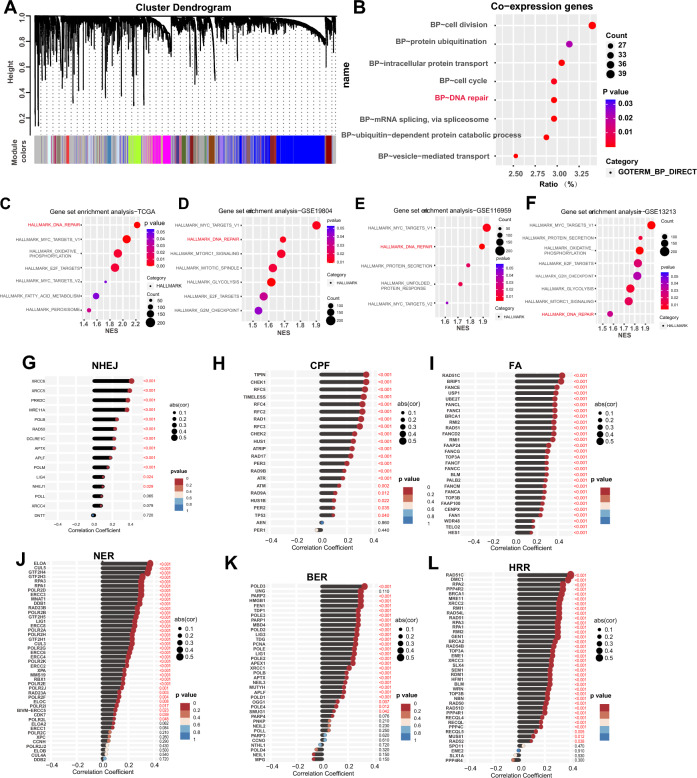
Functional enrichment analysis of HMGN1 in LUAD. (**A**) WGCNA cluster dendrogram showing co-expression gene modules in LUAD; the ‘brown’ module contains genes co-expressed with HMGN1. (**B**) Bubble diagram of GO enrichment analysis for HMGN1 co-expressed genes. (**C**–**F**) Bubble map of GSEA for HMGN1 from TCGA (**C**) and three GEO datasets (**D**–**F**). (**G**–**K**) Lollipop charts correlating HMGN1 with genes in different DNA repair pathways; CPF: check point factors; FA: fanconi anemia; NER: nucleotide excision repair; BER: base excision repair; NHEJ: non-homologous end joining; HRR: homologous recombination repair.

To investigate the function of HMGN1 in lung adenocarcinoma, we first explored the potential co-expression genes of HMGN1 using the weighted gene co-expression network analysis (WGCNA) followed by GO analysis. The network constructed on 1157 genes led to the identification of 100 modules comprising genes with similar expression patterns. GO analysis further pointed out that cell division, protein ubiquitination, intracellular protein, cell cycle, and DNA repair are the top five modules which were related to the expression of HMGN1 in LUAD (Fig. 4A and B). To further verify the physiological functions of HMGN1, Gene Set Enrichment Analysis (GSEA) analysis was then undertaken to investigate hallmarks associated with HMGN1 expression. The results confirmed the association of HMGN1 with DNA repair pathways (Fig. 4C and Supplementary 3F). Additionally, DNA repair were the top two gene sets with significant association with HMGN1 in multiple LUAD cohorts (Fig. 4D–F and Supplementary 3A). Notably, GSEA analyses of HMGN1 in several other cancer types have also emphasized the essential roles of HMGN1 in DNA repair (Supplementary 3B–E and G). DNA repair pathways primarily consist of six modalities: base excision repair (BER), nucleotide excision repair (NER), crypto-chrome/photolyase family (CPF), Fanconi Anemia DNA Repair Pathway (FA), non-homologous end joining (NHEJ), and homologous recombination repair (HRR). Correlation analysis indicated that HMGN1 expression was linked to all of these six DNA damage repair pathways, especially HRR (Fig. 4G–L).

### HMGN1 loss reduces the DNA repair response

**Figure 5:**
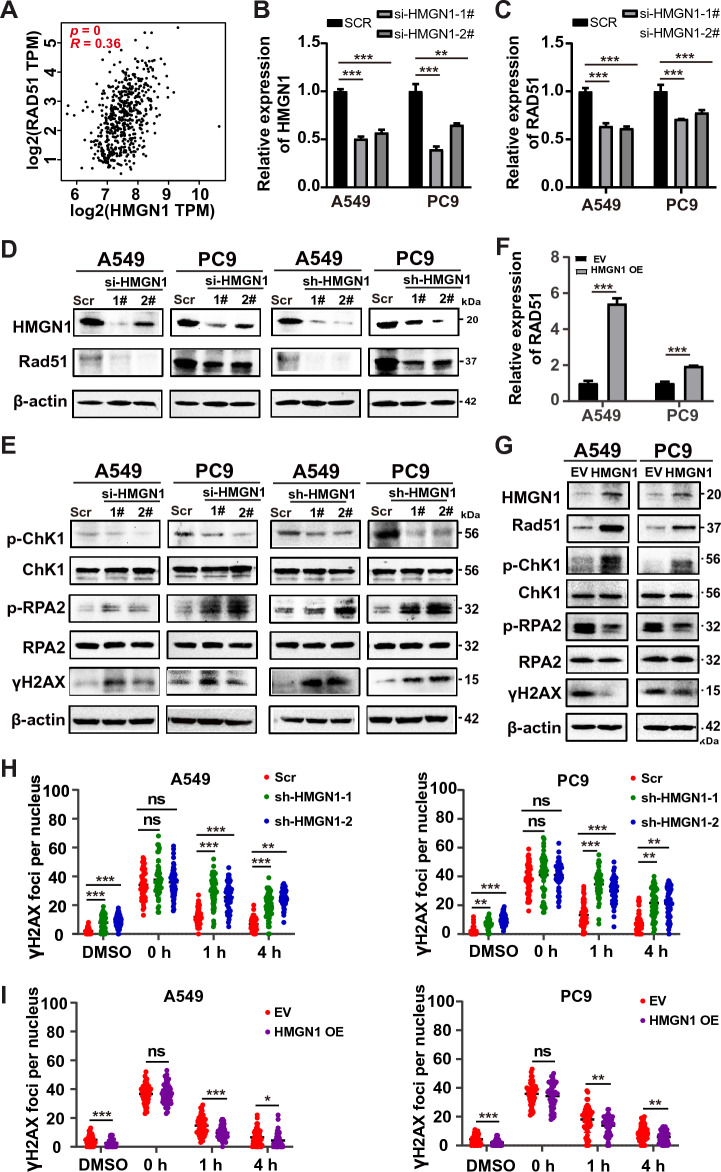
Impact of HMGN1 knockdown on DNA damage response in lung adenocarcinoma. (**A**) Pearson correlation analysis showing the correlation between HMGN1 expression and RAD51 expression in LUAD. (**B**, **C**) RT–qPCR analysis of HMGN1 silencing in A549 and PC9 cells and its effect on RAD51 expression. (**D**, **E**) Western blot analysis of Rad51, p-RPA2, γH2AX and p-Chk1 proteins following HMGN1 depletion. (**F**) RT–qPCR analysis of RAD51 expression upon ectopic expression of HMGN1. (**G**) Western blot analysis of Rad51, p-RPA2, γH2AX and p-Chk1 proteins following HMGN1 overexpression. (**G**) Quantification of Gamma-H2AX fluorescence foci from three experiments shown as mean ± SD. *p < 0.05, **p < 0.01,***p < 0.001, ns: not statistically significant.

Given that double-strand break (DSB) is the most lethal form of DNA damage and HMGN1 function in DSB repair remains largely unknown, above analysis prompted us to test whether HMGN1 is involved in DSB repair in lung adenocarcinoma cells. First, the mRNA expression of HMGN1 showed positive correlation with RAD51, a key recombinase in DSB repair, in the TCGA LUAD cohorts (Fig. 5A). Next, efficient depletion HMGN1 expression using siRNA was confirmed at the RNA and protein level in A549 and PC9 cells (Fig. 5B). We then assessed the effect of HMGN1 knockdown on Rad51 expression. The results showed that RAD51 expression was remarkably decreased at the mRNA and protein level (Fig. 5C). Importantly, p-CHK1, an essential component of the ATR-ChK1 pathway and DSB repair, was markedly reduced. At the same time, p-RPA2, a readout of DSB end resection in response to DNA damage, was remarkably accumulated after knocking down HMGN1 (Fig. 5D). In agreement with the western results for Rad51, p-CHK1, and p-RPA2, HMGN1 depletion led to robust accumulation of γH2AX, a classic DSB marker. Similar phenomenon was also seen in HMGN1 stably knockdown A549 and PC9 cells (Fig. 5D and E). On the contrary, HMGN1 overexpression promoted DNA damage response and reduced γH2AX expression (Fig. 5F and G). Furthermore, we have performed immunofluorescence staining for gamma-H2AX over time after the cells were treated with etoposide. The immunostaining results demonstrated that more γH2AX foci remained in HMGN1-depleted cells than HMGN1-proficient cells (Fig. 5H and I, Supplementary Fig. 4), suggesting reduced DSB repair capacity in the absence of HMGN1. In sum, these data suggest that HMGN1 inhibition attenuates the ATR-ChK1 pathway and subsequent Rad51-mediated recombination, thereby impairing DSB repair.

### HMGN1 depleted cells are more sensitive to HU treatment

**Figure 6:**
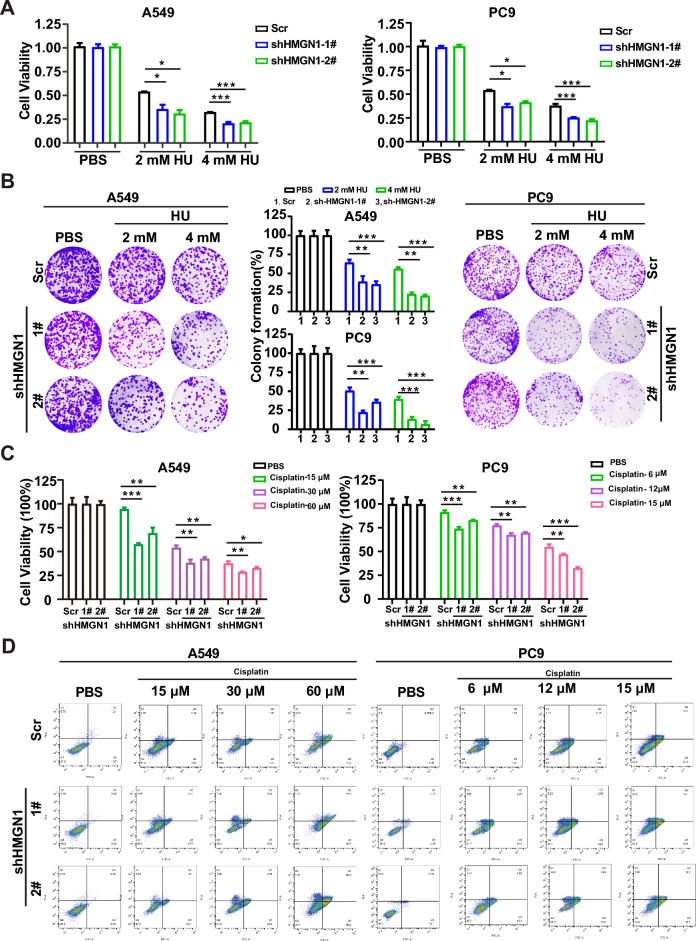
The effect of HMGN1 knockdown on HU or cisplatin sensitivity in lung adenocarcinoma. (**A**, **C**) Cell viability assays showing the effects of cisplatin and hydroxycarbamide (HU) on A549 and PC9 cells with HMGN1 knockdown. (**B**) Clonogenic assays of A549 and PC9 cells treated with varying concentrations of HU after HMGN1 knockdown. Surviving fraction percentages calculated relative to untreated cells. (**D**) Effect of HMGN1 depletion on cisplatin-induced cell apoptosis. *p < 0.05, **p < 0.01, ***p < 0.001, ns: not statistically significant (Student’s t test).

The indispensable role of HMGN1 in HRR prompted us to test the potential of HMGN1 inhibition to sensitize lung adenocarcinoma cells to DNA damage agents, which is frequently applied in clinical setting. First, we investigated the effect of HMGN1 knockdown on the sensitivity of hydroxyurea (HU). As shown in Fig. 6A and Supplementary fig 5, HMGN1-deficent A549 and PC9 cells were more much more sensitive to HU treatment, which was consistent with the role of HMGN1 in the ATR-ChK1 pathway. Moreover, long-term colony formation assays demonstrated that HMGN1 could protect the cells from HU-induced toxicity (Fig. 6B). To explore the chemosensitizing potential of targeting HMGN1, we treated A549 and PC9 cells with a range of concentrations of cisplatin to assess cell survival in the absence of HMGN1. As displayed in Fig. 6C, HMGN1 silencing could enhance the cytotoxic activity of cisplatin, a commonly used chemotherapeutic drug in the clinic. Concomitantly, a markedly increase was seen in HMGN1-deficient cells compared with HMGN1-proficient cells (Fig. 6D).

## Conclusion

Lung cancer, as the most common cause of cancer-related deaths globally, with lung adenocarcinoma (LUAD) being its most common histological subtype, continue to present significant challenges. Despite advances in diagnosis and treatment in the past decades, there remains a critical need for novel prognostic markers and therapeutic strategies for LUAD, which is characterized by an extremely poor 5-year survival rate. Aberrant epigenetic regulation, alongside genetic alterations, has been implicated in every step of lung cancer. Among epigenetic factors, the High Mobility Group Nucleosome Binding (HMGN) family, known for its specific interaction with nucleosomes in chromatin, particularly in enhancers and promoters, is crucial in establishing cell-type-specific gene expression programs. Although HMGNs’ involvement in various cancers has been noted, their roles in LUAD remain unclear.

This study utilized publicly available datasets and bioinformatics approaches to comprehensively analyze the expression and prognostic value of HMGNs in LUAD. We focused on HMGN1 due to its significant differential gene expression. Our results suggest HMGN1’s potential as a prognostic marker for distinguishing LUAD patients from healthy individuals. Functional enrichment analysis and cellular experiments emphasized HMGN1’s critical role in the DNA damage response, particularly in Homologous Recombination (HR).

We observed that HMGN1 were highly expressed in LUAD tissues compared with normal tissues at the RNA and protein level, while HMGN3/5 were expressed at a lower level in the tumor tissues compared to normal controls. Mutation profile and promoter methylation analysis indicate that both copy number variation and promoter methylation might contribute the dysregulation of HMGNs, though the underlying mechanisms lead to these alterations remain under investigation. Interestingly, HMGNs show a relatively low mutation rate in lung adenocarcinoma, this phenomenon has been observed for a number of epigenetic factors. We speculated that HMGN1 might be too critical to be mutated due to its importance in modulation of chromatin structure and histone modifications.

To identify potential signaling pathways regulated by HMGN1, we constructed a module-centric co-expression network based on weighted gene co-expression network analysis (WGCNA) and analyzed the possible functions of the correlated gene set by GO analysis. The results highlighted that cell division, protein ubiquitination, intracellular protein, cell cycle, and DNA repair were significantly associated with HMGN1 expression in LUAD. Moreover, functional enrichment analysis by GSEA confirmed that HMGN1 was highly involved in the regulation of the DNA repair signaling, which is in line with the previous findings in UV and irradiation-treated cells. Since the roles of HMGN1 in specific DNA repair pathways are not well characterized. We set to correlate the specific DNA repair pathways with HMGN1 expression and found that HRR and NER were the top two pathways among the six classic DNA repair pathways. Subsequent in vitro experiments using PC9 and A549 cells confirmed that silencing HMGN1 attenuated HR repair capacity.

Indeed, several studies suggest that HMGN1 manifests anti-tumor effects by promoting DNA repair and genome stability^35^. Interestingly, high HMGN1 expression correlates with poor prognosis in LUAD, despite its role in promoting DNA repair. Two recent studies have demonstrated that HMGN1 acts chromatin architectural protein to compete with histone H1 for nucleosome binding sites^7,36^. HMGN1 supports repair of DNA lesions, it would be expected that histone H1 will have an opposite effect on DNA damage repair. Indeed, a number of studies suggest that histone H1 has an inhibitory effect on repair of DNA lesions^36,37^. Another investigation indicates that histone H1 suppresses repair of DSB in vitro when present in high concentrations^38^. The above observations suggest that the balance between the relative amounts of HMGN1 and histone H1, as well as their chromatin binding abilities, may be essential for the regulation of the cellular response to DNA damage^39^. Therefore, how HMGN1 ultimately participates in the progression of lung adenocarcinoma remains to be explored.

However, there are some limitations to this study. First, although our experimental data showed the functional connection between HMGN1 and the DNA damage response pathway, the underlying mechanisms leading to the down-regulation of RAD51 and CHK1 phosphorylation remain to be determined. Perturbation of HMGN expression might interfere with the expression of some DNA damage response genes including RAD51 on the basis of the preferential association of HMGN proteins with chromatin regulatory sites including enhancers and promoters. Second, only one pathway of DNA repair has been verified, and other enrichment pathways need to be further addressed.

In summary, our systematic analysis positions HMGN1 as a diagnostic and prognostic marker for lung adenocarcinoma. Moreover, HMGN1 holds great potential as an epigenetic therapeutic target given that HMGN1 plays critical roles in the DNA damage response, especially in HRR. Given the pivotal role of HMGN1 in peri-tumor infiltration of lymphocytes, combining HMGN1 targeting with immunotherapy might achieve better therapeutic outcomes for lung adenocarcinoma.

### Supplementary Information

Supplementary Figures.

## Data Availability

The datasets generated during and/or analyzed during the current study are available in the TCGA repository, https://tcga-data.nci.nih.gov/tcga; UALCAN repository, http://ualcan.path.uab.edu; Human Protein Atlas repository, https://www.proteinatlas.org; cBioPortal repository, https://www.cbioportal.org; KMplot repository, https://kmplot.com; Timer repository, https://cistrome.shinyapps.io/timer; GEO repository, http://www.ncbi.nlm.nih.gov/geo/.

## Abbreviations

LUAD: Lung adenocarcinoma
HMGN: The high mobility group nucleosome-binding protein
CNV: DNA copy number variation
ROC: The receiver operating characteristic curve
AUC: Area under the curve
HR: Hazard ratio
OS: Overall survival
PFS: Progression-free survival
DFS: Disease-free survival
DSS: Disease-specific survival
HU: Hydroxyurea
