# Supplementary material for: HMGN1 loss sensitizes lung cancer cells to chemotherapy
Source: Sci Rep. 2024 May 6;14:10386. doi: 10.1038/s41598-024-60352-8 (PMC11074128; doi:10.1038/s41598-024-60352-8)

**Supplement figure 1. Abnormal expression of HMGN family in lung adenocarcinoma.** (A) Expression comparison of HMGN in lung adenocarcinoma tumor tissues and unmatched normal tissues from different databases. (B) Representative immunohistochemistry images of HMGNs in LUAD and normal lung tissues (Human Protein Atlas), with upper panel showing normal lung tissues and lower panel showing LUAD. (C) RNA expression of HMGN1 in various cancer types (TIMER database). *p<0.05, **p<0.01, ***p<0.001, ns: not statistically significant.

**Supplement figure 2. Prognostic value of HMGN1 in LUAD patients.** (A) ROC analysis of HMGN1 expression in different lung cancer subtype. (B) Kaplan–Meier analysis of HMGN1 expression linked to progression free and disease-free survival in LUAD from the TCGA database. (C) Kaplan–Meier analysis showing that association of HMGN1 expression with overall survival and first progression survival in LUAD from the Kaplan–Meier plotter website.

**Supplement figure 3. Functional enrichment analysis of HMGN1 in lung adenocarcinoma.** (A) Bubble map showing the gene set enrichment analysis of HMGN1 from GSE11969. (B-E) Bubble maps showing the gene set enrichment analysis of HMGN1 in different cancer types from TCGA. (F) GSEA maps showing highly enriched DNA repair related pathways associated with high HMGN1 expression in LUAD. (G) Heat map displaying expression DNA repair-related genes in relation to HMGN1expression level.

**Supplement figure 4. HMGN1 promotes DNA damage response.**

(A-D) Gamma-H2AX immunofluorescent staining in HMGN1-deficient or overexpressed cell lines, respectively. The indicated cells were treated with etoposide (40 nM) for 1 h. Cells were then washed, shifted to fresh medium (time 0,1,4h), and harvested at the indicated time points for immunostaining with Gamma-H2AX antibody. Scale bar,50μm.

**Supplement figure 5. HMGN1 silencing enhances HU-induced apoptotsis.** Annexin V apoptotic assay indicates that HMGN1 silencing facilitates HU-mediated apoptosis. SCLC cells were treated with PBS control, 2 mM HU for 2 hours, 4 mM HU for 4 h. After treatment, Annexin V apoptotic assay was performed by flow cytometry.

**original blots/gels are presented in Supplementary Figure 6**.The changes of the proteins (β-actin,Chk1,RPA2,p-Chk1,p-RPA2) gels detected after HMGN1 knockdown in Figure 5D and 5E. Full length representative western blot membranes are shown, with probed target protein superimposed onto colorimetric images of protein ladders. Numbers on ladder represent known kilodaltons (kDa) flanking protein of interest.

**original blots/gels are presented in Supplementary Figure 7**. The changes of the proteins (Rad51,HMGN1,γH2AX) gels detected after HMGN1 knockdown in Figure 5D and 5E.

**original blots/gels are presented in Supplementary Figure 8.** The changes of all the proteins (β-actin,Chk1,RPA2,p-Chk1,p-RPA2, Rad51,HMGN1,γH2AX) detected after HMGN1 overexpression in Figure 5G. Full length representative western blot membranes are shown, with probed target protein superimposed onto colorimetric images of protein ladders. Numbers on ladder represent known kilodaltons (kDa) flanking protein of interest.


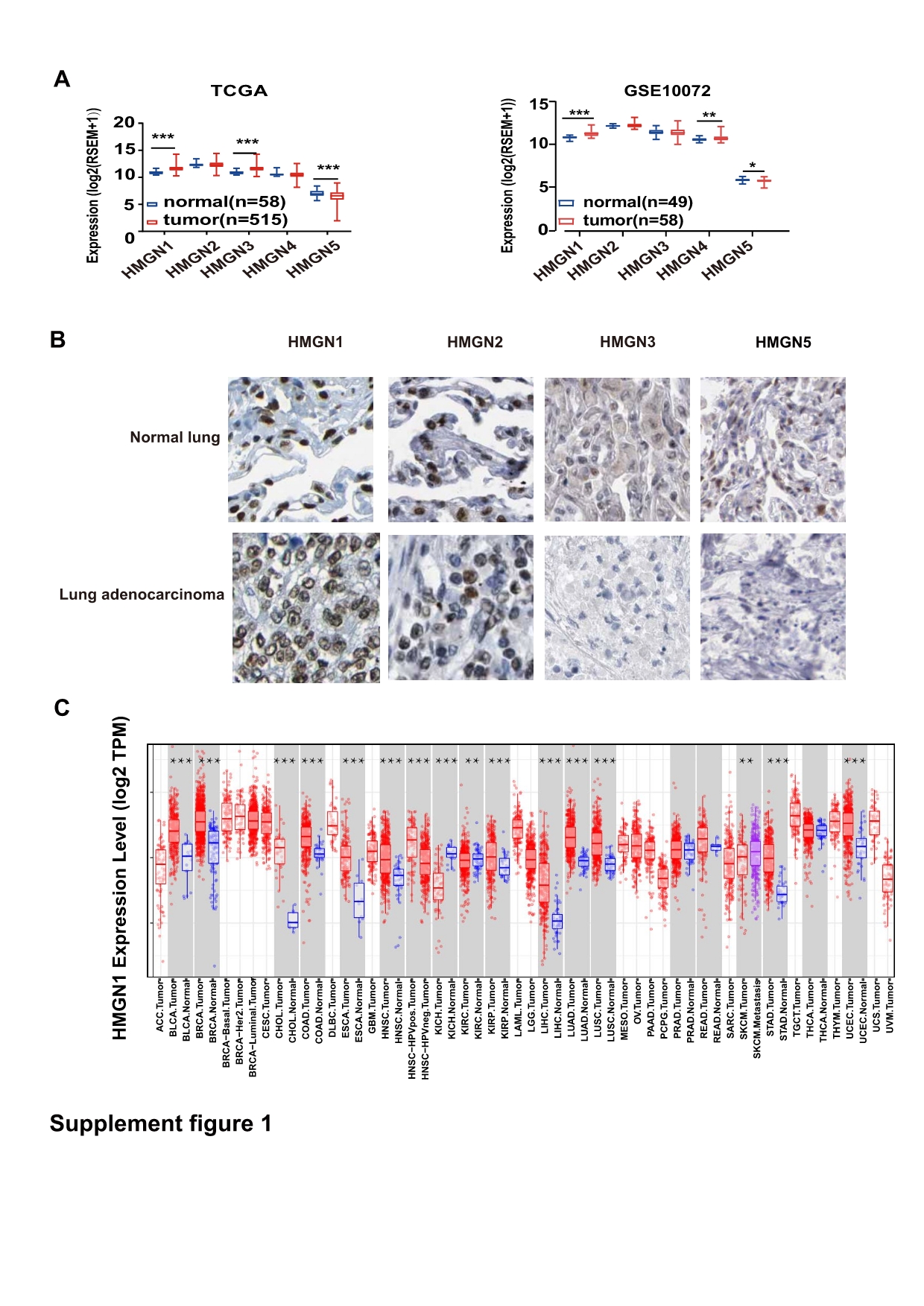


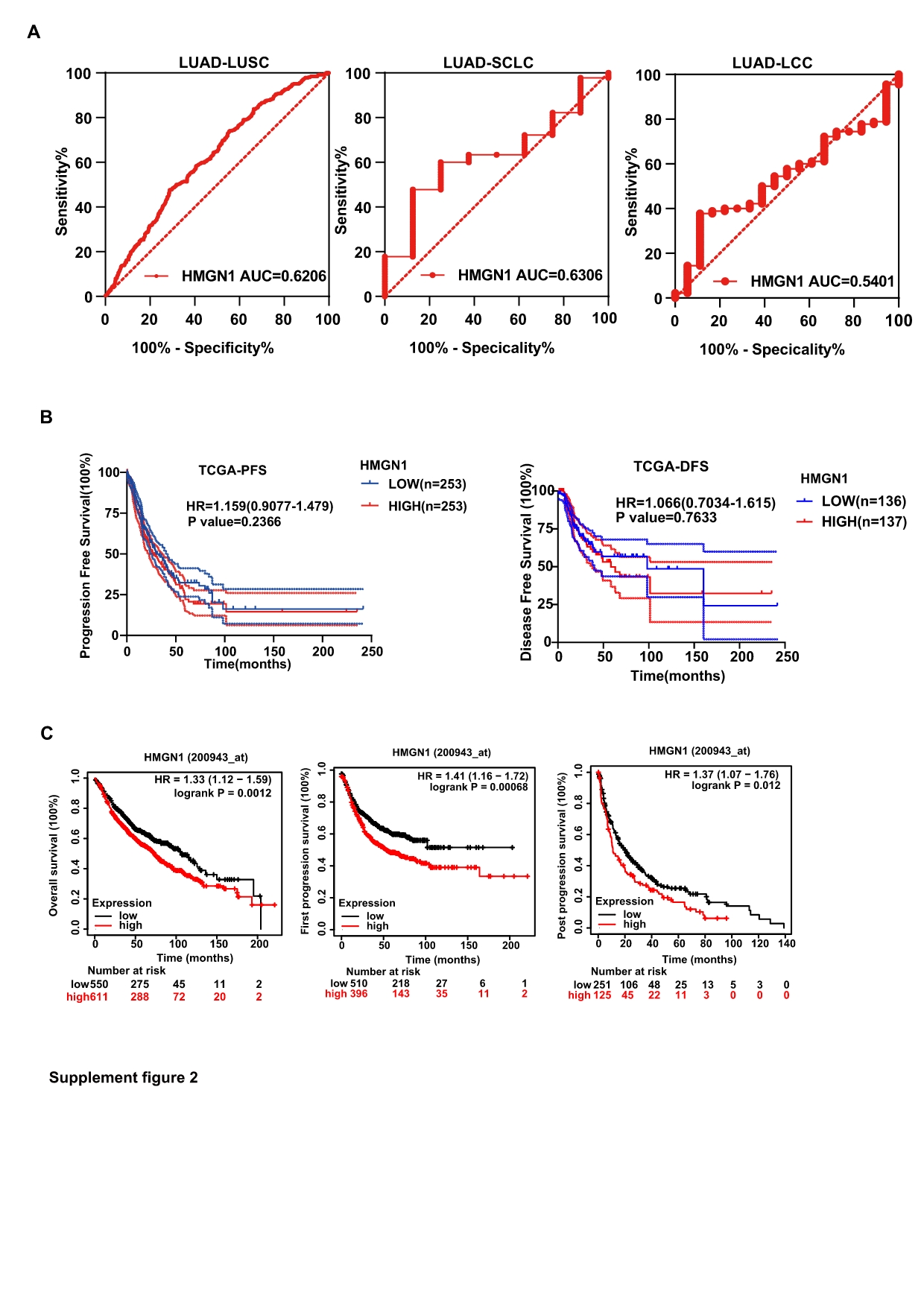


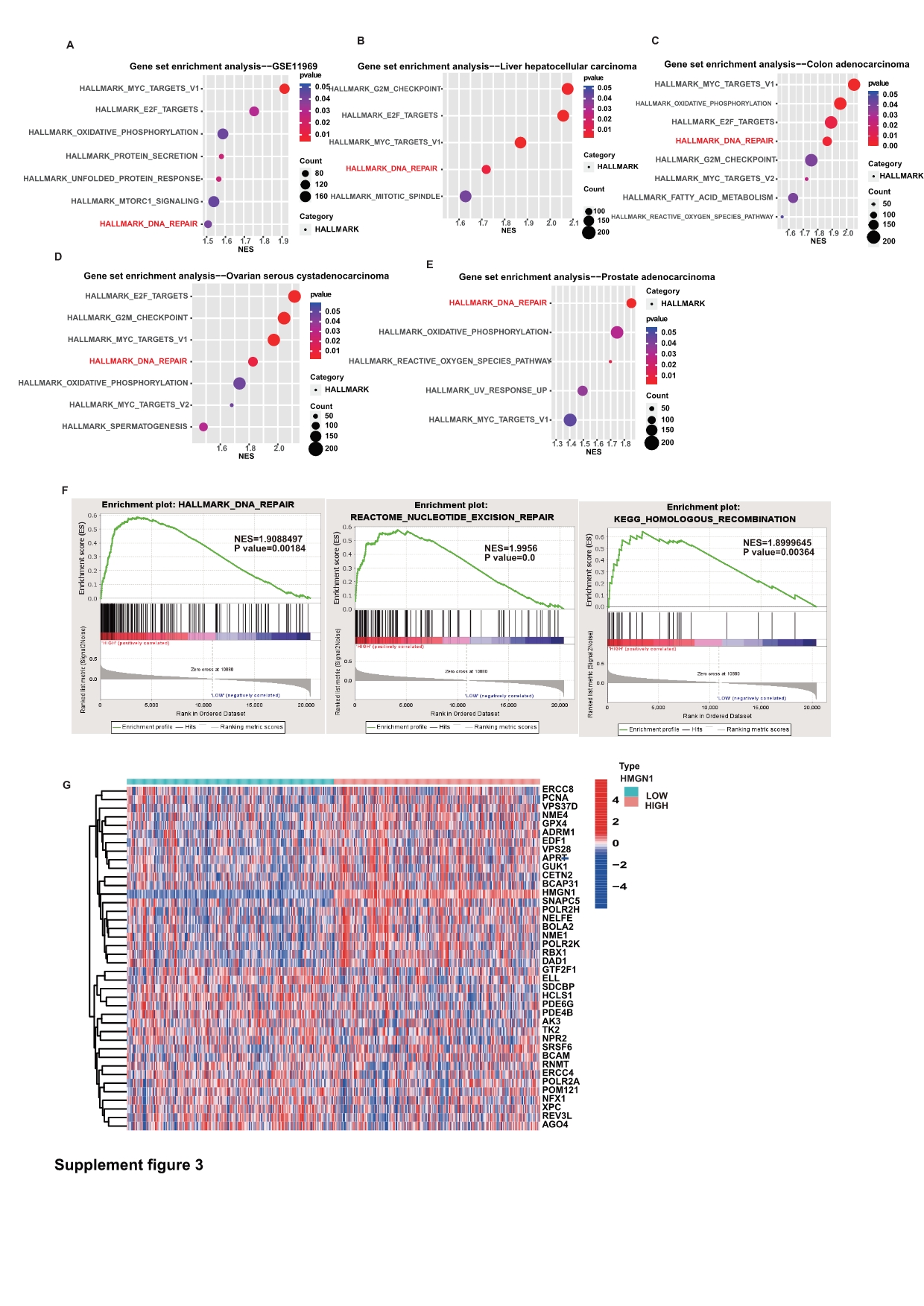


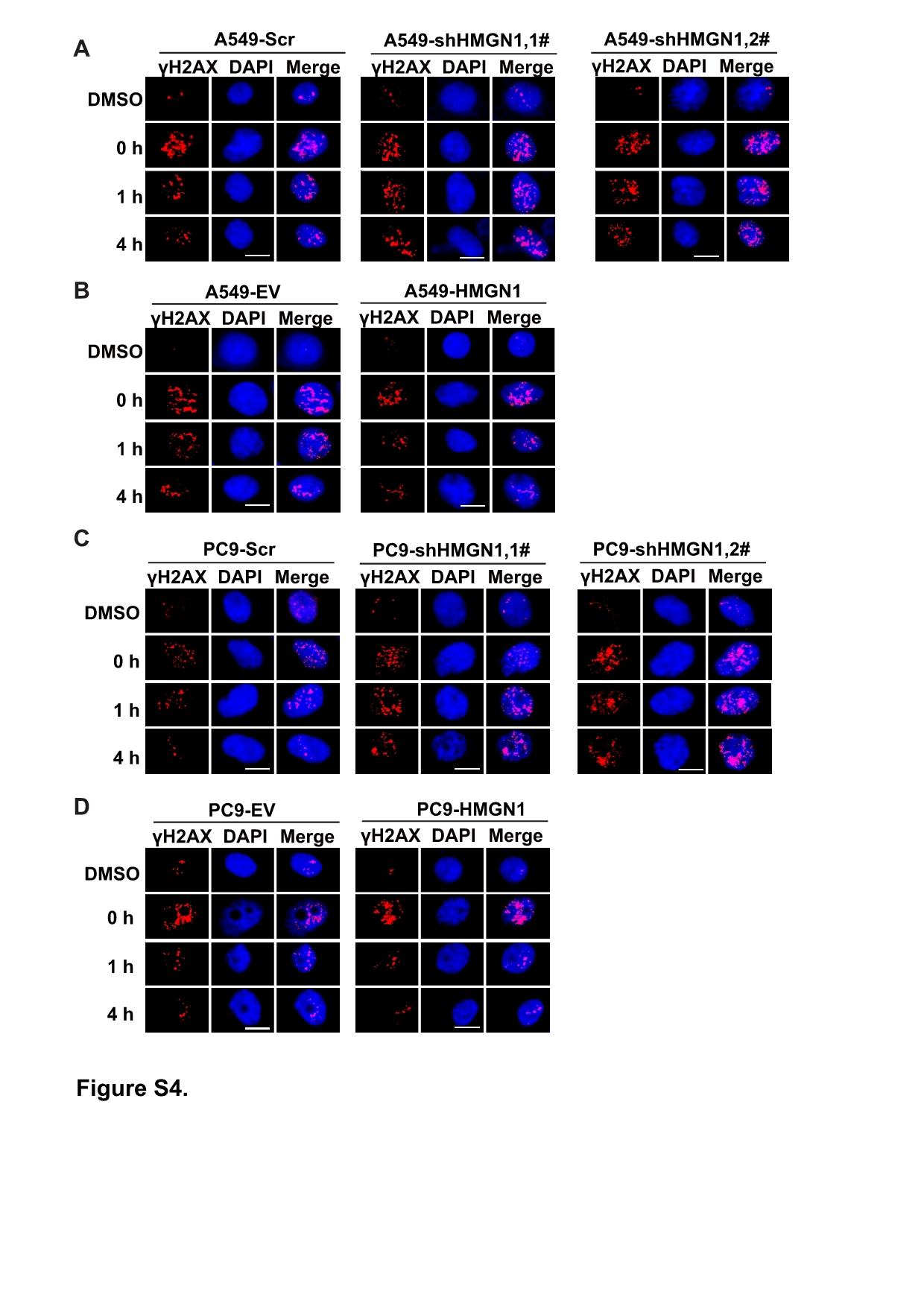


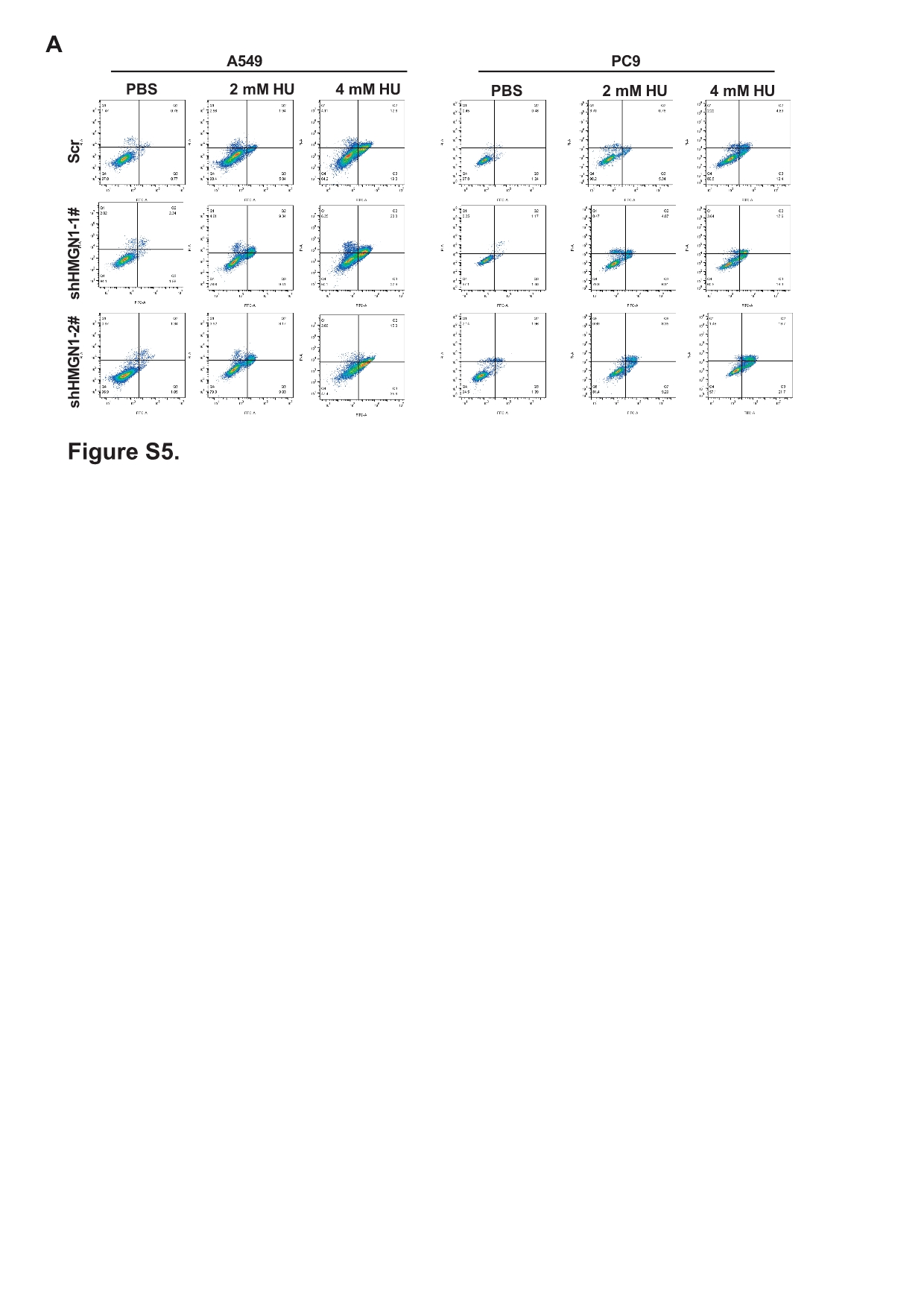


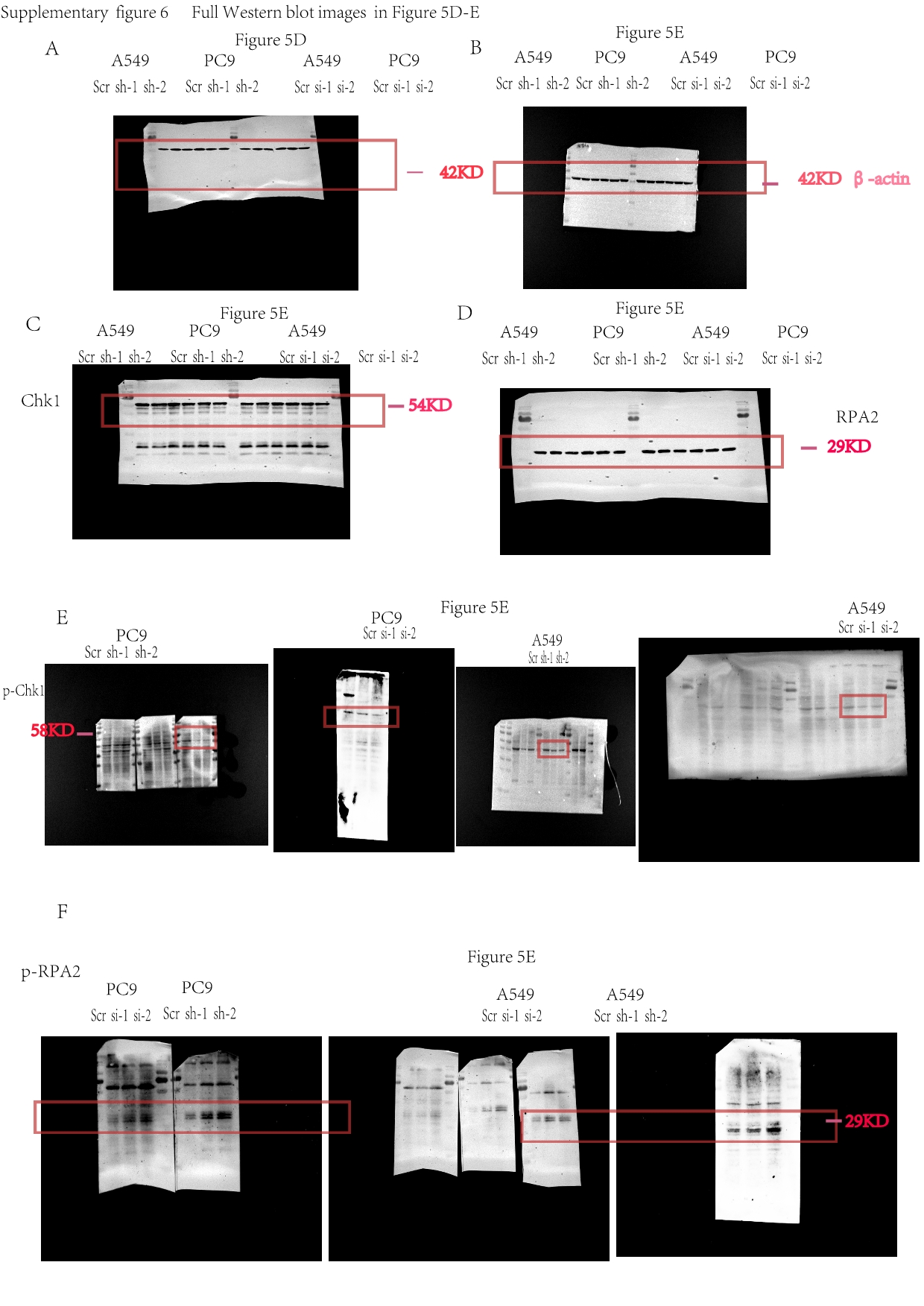


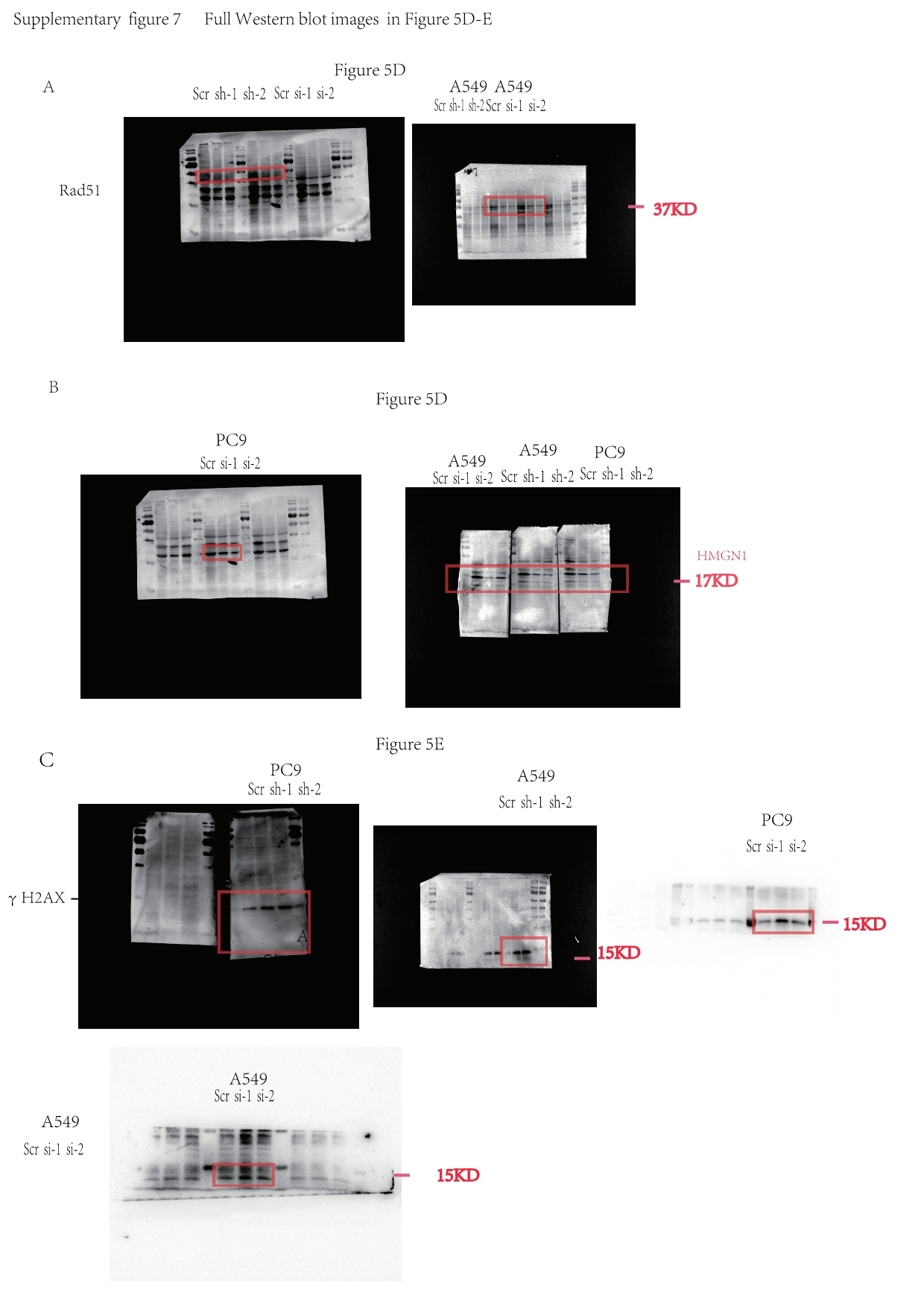


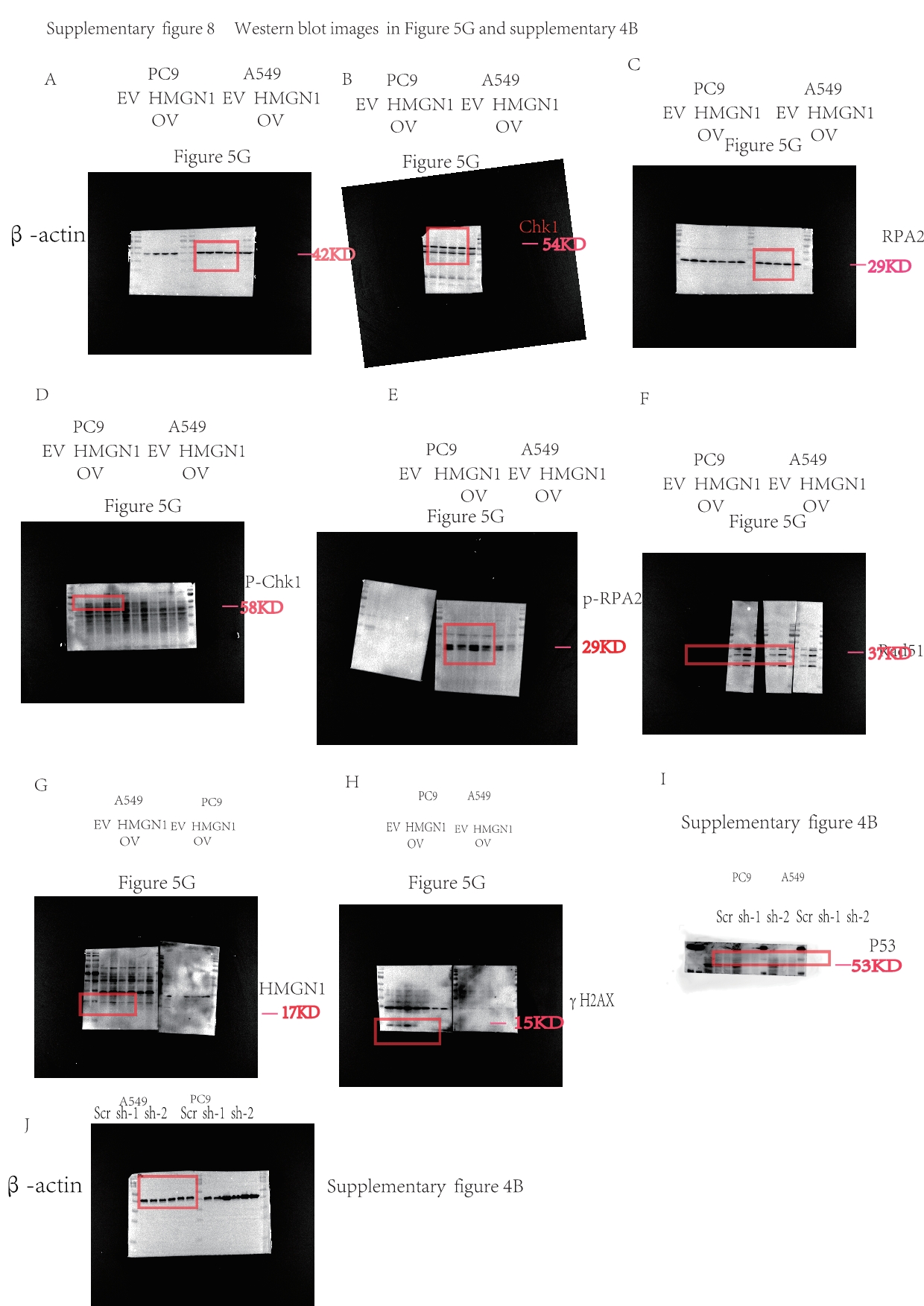

Supplement: Supplementary file 1 — Supplementary Figures. [file 41598_2024_60352_MOESM1_ESM.docx]
